# Cases of a Dead Fœtus, Retained in Utero, over Four Months; and Cases of Prolonged Pregnancy

**Published:** 1875-06

**Authors:** A. R. Kilpatrick

**Affiliations:** Navasota, Texas


### CASES OF A DEAD FŒTUS, RETAINED IN UTERO, OVER FOUR MONTHS; AND CASES OF PROLONGED PREGNANCY.

BY A. R. KILPATRICK, M. D., NAVASOTA, TEXAS.

Mrs. J. S. had been married thirteen years, and had enjoyed good health, but was never pregnant till now; 32 years of age.

When she was advanced to about the fourth month of gestation, and thought she had felt the motions of the fœtus, it was killed by an accident. She was preparing to move to another house, and while standing on a small table, engaged in taking down window curtains, she fell and struck her abdomen with violence on the corner of a Saratoga trunk. This caused quite a shock, much pain, some hemorrhage, with the usual prodromata of miscarriage, but by timely assistance this was averted. After recovery, she made the intended change of residence, where I saw her afterwards for the first time, by her husband's request, a few weeks before her expected confinement.

When she entered the parlor, being habited in close-fitting apparel, she was so slender, and looked so little like a woman approaching the full term of gestation, that the idea occurred to me that she was either self-deceived, or else was far wrong in her calculations.

Having been annoyed on former occasions by women who wished to be "as ladies like to be who love their lords," I thought here was a case of that kind, and expressed my surprise and doubts to her husband, but he assured me she was pregnant and near full term, which she also affirmed. I concluded to wait for further developments.

In a few weeks I was called on to attend her in labor. Every thing proceeded as in ordinary cases of primiparæ, but the uterus was not much over the usual size at the fourth or fifth month, although she averred that she had come to full term.

The labor was slow and she complained very much. There was very little liquor amnii and it passed off early. There was some floculi in it, and it was soon evident that the fœtus was dead. After she had been in labor altogether fifteen hours, the delivery was accomplished, revealing a dead male, in an advanced state of decomposition. The epidermis was nearly all rubbed off, the fœtus was dark and offensive; the umbilical cord, although much decayed, was not broken; the placenta came with the child, and was disorganized.

There was very slight loss of blood, and the uterus contracted well. There was a slight secretion of milk. She recovered as well, and as rapidly, as any woman after natural labor at full term.

She insisted that she had felt the motions of the fœtus repeatedly, and even during labor. This shows how her mind, and feelings, had been subordinated to her imagination, and her strong maternal desires and impulses.

A very striking feature of the case is that she should continue in a healthy condition, after recovering from the injuries inflicted by the fall, and carry a dead and putrescent fœtus four months.—Another is that the labor should come on at the regular time of nine months, after conception before the uterus had reached its full expansion, or the cervix had been obliterated. Why should it not have taken place sooner or later than the ninth month? Authors mention other cases like this, but we can only speculate on the causes and reasons. In extra-uterine fætation, a great number of cases are recorded, where the dead fœtus was retained, without injury to the woman, from a few months to fifty-six years.

Several years ago a case of gestation, prolonged to ten months, came under my observation, and is the only one I have seen. The patient was a very intelligent lady, living in the country; had borne three other children, and suffered one miscarriage. Although of large frame, she was of nervous temperament, and her general health rather delicate. I am satisfied there was no effort on her part to deceive me, and her having borne children rendered her more competent to judge of the time of conception, and forecast the period of anticipated labor.

As she was a near neighbor, our families were in constant intercourse, and the time looked forward to with some interest. It came, and passed on, and on, till fully another month elapsed before labor came on.

I delivered her of a female child which weighed eighteen pounds, and was by far the longest I ever saw. Although the labor was natural, and not over four hours, yet the child was dead, and seemed to have been dead two or more days; although there were no symptoms noticed by her which indicated the fact.

There are many cases recorded by authors where gestation has been prolonged to three hundred and even as high as three hundred and sixteen days.

